# Validation of reference genes for quantitative RT-qPCR studies of gene expression in Atlantic cod (*Gadus morhua l*.) during temperature stress

**DOI:** 10.1186/1756-0500-4-104

**Published:** 2011-04-05

**Authors:** Ingrid A Aursnes, Anne L Rishovd, Hans E Karlsen, Tor Gjøen

**Affiliations:** 1 Department of Pharmaceutical Biosciences, School of Pharmacy, University of Oslo, PO Box 1068 Blindern, 0316 Oslo, Norway; 2 Institute of Biology, University of Oslo, Blindern N-0316, Norway

## Abstract

**Background:**

One important physiological response to environmental stress in animals is change in gene expression. To obtain reliable data from gene expression studies using RT-qPCR it is important to evaluate a set of possible reference genes as normalizers for expression. The expression of these candidate genes should be analyzed in the relevant tissues during normal and stressed situations. To find suitable reference genes it was crucial that the genes were stably expressed also during a situation of physiological stress. For poikilotermic animals like cod, changes in temperature are normal, but if the changes are faster than physiological compensation, the animals respond with typical stress responses. It has previously been shown that Atlantic cod show stress responses when elevation of water temperature is faster than 1 degree/day, for this reason we chose hyperthermia as stress agent for this experiment.

**Findings:**

We here describe the expression of eight candidate reference genes from Atlantic cod (*Gadus morhua l*.) and their stability during thermal stress (temperature elevation of one degree C/day for 5 days). The genes investigated were: Eukaryotic elongation factor 1 alpha, *ef1a*; 18s ribosomal RNA; *18s*, Ubiquitin conjugate protein; *ubiq*, cytoskeletal beta-actin; *actb*, major histcompatibility complex I; MHC-I light chain, beta-2 -microglobulin; *b2m*, cytoskeletal alpha-tubulin; *tba1c*, acidic ribosomal phosphoprotein; *rplp1*, glucose-6-phosphate dehydrogenase; *g6pd*. Their expression were analyzed in 6 tissues (liver, head kidney, intestine, spleen, heart and gills) from cods exposed to elevated temperature and compared to a control group. Although there were variations between tissues with respect to reference gene stability, four transcripts were more consistent than the others: *ubiq*, *ef1a*, *18s *and *rplp1*. We therefore used these to analyze the expression of stress related genes (heat shock proteins) induced during hyperthermia. We found that both transcripts were significantly upregulated in several tissues in fish exposed to increased temperature.

**Conclusion:**

This is the first study comparing reference genes for RT-qPCR analyses of expression during hyperthermia in Atlantic cod. *ef1a, 18s, rplp1 *and *ubiq *transcripts were found to be well suited as reference genes during these experimental conditions.

## Findings

A good reference gene should be expressed in detectable amounts without too much variation between tissues. We have investigated the expression stability of eight genes, commonly used as reference genes, for internal control in experiments. Five of these genes (Eukaryotic elongation factor 1 alpha, *ef1a*; 18s ribosomal RNA; *18s*, Ubiquitin conjugate protein; *ubiq*, cytoskeletal beta-actin; *actb*, acidic ribosomal phosphoprotein; *rplp1*, are in common with the previous study [1] and three genes have not been tested before in cod. The experimental conditions in this setting were tissue samples from wild Atlantic cod exposed to hyperthermia.

The chosen genes have different tasks in the cell; *ubiq *is a protein involved with degradation of cellular proteins, *tba1c *and *actb *are both parts of the cytoskeleton. *b2m *is part of the MHC I complex on cell surface. *rplp1*, *ef1a *and *18s *are all involved in protein synthesis. Acidic ribosomal proteins are conserved phosphoproteins involved in the translation process and may therefore be co-regulated with *ef1a *[2]. Glucose-6-phosphate dehydrogenase (*g6pd*) is a cytosolic enzyme that takes part in the pentose phosphate pathway. In this selection of genes it is possible that some co-regulation may occur, and it is important to keep this in mind when evaluating the geNorm results. The stress response induced by hyperthermia was confirmed by expression analysis of two heat shock proteins, HSP70 and HSP90AA.

## Materials and methods

Atlantic cod (size 300-500 g) were obtained from fish traps in the Oslofjord and kept in 0.3 m^3 ^tanks with running seawater. The animals were divided in two experimental groups containing five wild fishes in each group. The fish in the heat stress group were kept in a pool of water in which the temperature was elevated with 1 degree each day, during a period of 5 days. The starting temperature was 11°C and after five days of heating the water temperature was 17°C. The fish in the control group were kept in a similar tank with stable temperature to minimize the differences in general stress from animal housing.

Fish were killed after 5 days and tissue samples were taken from heart, spleen, head kidney, small intestine, gills and liver and put directly into RNA-later and frozen (-20°C) until further analysis. All animal experiments were conducted in accordance with the Norwegian Animal Welfare Act of 1977, and the Regulation of Animal Experimentation of 1996.

### RNA extraction

The tissue samples were mixed with beads and homogenized by a rapid vibration in a Precellys 24 (Bertin Technologies, France). Total RNA were extracted from the tissue samples using the RNeasy Mini Kit with on column RNase-free DNase set (Qiagen). The protocol was according to the manufacturer. Duplicates of cDNA were made from 2 ug total RNA using High Capacity RNA-to-cDNA Master Mix, 4 × 96-Well Plate (ABI) and after the manufacturer's protocol.

### Genes and primers

Atlantic cod ESTs representing the genes to be analyzed (see Table 1) were obtained by tblastn searching the respective protein sequences from the TIGR zebrafish (Danio rerio) [3] against Genbank [4] or Codgene [5]. The chosen target genes were heat shock protein 70 (*hsp70*) and heat shock protein 90 alpha (*hsp90)*.

**Table 1:** Primers for RTqPCR used in the present study.

Gene	Accesion #	Primer forward	Primer reverse	**E**.	Amplicon size
*rplp1*	EX741373	tccaaaccctaaaatccaaca	tggaggatcagagcagagtaaa	1,89	77
*actb*	AJ555463	acaccgtgcccatctacg	ccaagtccagacggaggat	2,05	60
*b2m*	acc386599	gagcccaacaccctgatct	gctcgatggtgatctctgg	2,11	61
*ef1a*	DQ402371	caggtcatcatcctgaacca	atccaggactggggcatag	2,02	60
*g6pd*	EX741924	acactctgcaccagggagtc	ccactgctgcaccacatc	1,88	98
*tba1c*	EL616694	ctccaccaggaactacagtgg	tagactggtgcccaactggt	2,10	69
*ubiq*	EX735613	cattgagccttccctcagaa	ttgcggcagatcatcttgt	1,98	63
*18s*	AJ427629	tgtgccgctagaggtgaaatt	gcaaatgctttcgctttcg	2,03	61
*hsp70*	ES478099	ggagttcaagcggaagttca	agcctcctcaaagccctct	1,87	60
*hsp90*	ES783928	tcctccgatactacacctcca	gcgagacacgtagtccttga	1,98	65

Reference and *hsp's *gene primer sequences, amplicon sizes, amplification efficiency values and accession # for the PCR analyses in the present study.

### Primer design

Primers (Table 1) were designed using the Roche Diagnostics primer design software and synthesized by InVitrogen (Carlsbad, USA). Primer efficiencies(E) were calculated from 10-fold dilution series to make standard curves from with E was calculated according to the formula E = 10^-1/slope^. The specificity of the primer sets used was confirmed by the presence of a single band of correct size on gel electrophoresis in addition to the presence of a single peak in the dissociation curve analysis.

### RT-qPCR

RT-qPCR was performed in duplicates in 96-well plates on Lightcycler 480 (Roche Diagnostics). SYBR Green Master Mix with cDNA made from 2 ug RNA and diluted 1:50 before added to the PCR reaction, except for *18s *which were used in a 1:1000 dilution. Crossing point values (Cp) values were obtained from the RT-qPCR instrument by employing the second derivative maximum method of LightCycler480 (Roche Diagnostics). The general guidelines for RT-qPCR experiments called "MIQE[6].

### Analyzes reference genes

A number of mathematical models have been developed to strengthen the normalization with reference genes. Examples of these are geNorm [7], Normfinder [8] and Bestkeeper [9]. GeNorm is restricted to reference gene analysis and eliminates the worst gene in a set of genes according to their expression stability. GeNorm perform a pair-wise variation of each combination of the genes and the highest expression stability gives the lowest M value. The least stable gene is eliminated until the two most stable genes remains. Instead of analyzing the whole data set, Normfinder focuses on the expression variations both between treatment groups and inside one group, in a model-based approach of mixed linear effect modeling. And finally, Bestkeeper analyze the variability of the dataset and also compare all genes against each other in pair-wise correlations, and also pair-wise correlation against the Bestkeeper index. Bestkeeper also have the ability to do a target gene analysis.

The Relative Expression Software Tool (REST) [10] was used to compare the hepatic expression of two target genes (*hsp70 *and *hsp90) *in the two groups relative to each of the reference genes.

## Results

**Figure 1:**
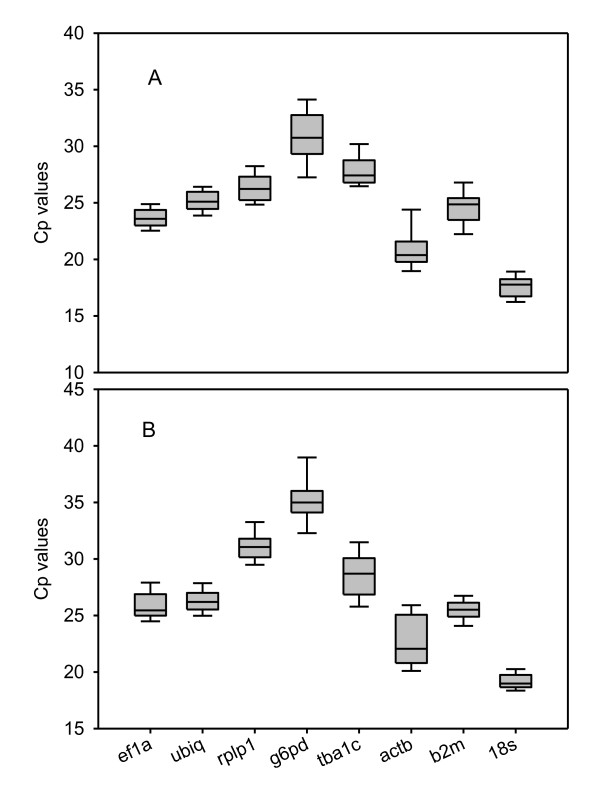
**Expression level of reference genes across tissues**. Expression level (Cp range) of candidate reference genes in various tissues (liver, head kidney, intestine, spleen, heart and gills) from Atlantic cod under control (A) and hyperthermia (B) conditions. The data are displayed as mean (line), 5^th^/95^th ^percentile (box) and range (whiskers). n = 5.

Figure 1A and 1B show box plots of raw Cp values displaying mean and 95% confidence intervals of the 8 candidate genes across all tissues in both control (A) and thermal stress group (B). The transcripts with lowest variation in control were *ef1a*, *ubiq *and *18s *(range 3, 8 for all 3). The other transcripts all had a Cp range above 5. In the stress group, *18s*, *ubiq *and *actb *were the only 3 genes with Cp-range below 5. *18s *and *ubiq *were therefore the only genes with low level of variance in both groups. To analyze the data further, we used the tree software packages developed for analysis of reference gene stability; geNorm, Normfinder and Bestkeeper. These programs use different algorithms to determine the most stable reference gene, and should be used together for identification of the most stable genes or gene combinations.

### GeNorm

**Figure 2:**
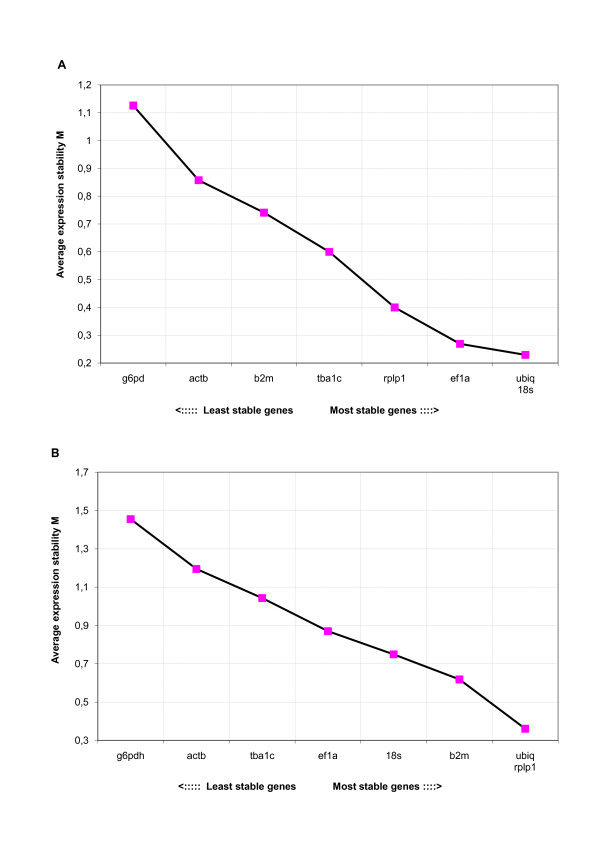
**Expression stability and ranking of reference genes by GeNorm software**. Ranking of the reference genes stability in pooled datasets from all six tissues according to the *geNorm *software. 2 A represent the result from the control group and 2 B show the expression stability in the heat stress group. A low average M-value indicates more stable expression.

When expression levels in tissues were analyzed separately in both treatment groups, the genes in the control group in general displayed higher stability (M) compared to the stress group. The results from analyzing data from all tissues revealed that *ubiq *was most stable in both control and stress group (Figure 2A and 2B, Table 2). In addition, *ubiq *was ranked as the best reference gene in at least one of the treatment groups in all tissues analyzed. The results from all individual tissues are summarized in Table 2. Genorm also calculates the pair wise variation between two sequential normalization factors (reference genes) NF_n _and NF_n+1_, reflecting the effect of including more than 2 reference genes in the normalization. High NF_n _/NF_n+1 _value mean a significant effect of including an additional gene in the calculations. Additional file 1 shows these ratios for inclusion of additional genes were lower for control (Panel A) than for the stress group (Panel B). In the control group 2 reference genes were sufficient (V2/3 < 0.15) whereas in the stress group a more reliable normalization will be obtained by inclusion of more reference genes, as expected. For practical purposes, 3 reference genes will normally be sufficient [7].

**Table 2:** GeNorm ranking of the reference genes stability in all investigated tissues,.

	Best gene		2^nd ^best		3^rd ^best		4^th ^best	
	Stress	Control	Stress	Control	Stress	Control	Stress	Control
Liver	*rplp1/ef1a*	*rplp1/ubiq*	*18s*	*tba1c*	*g6pd*	*g6pd*	*actb*	*actb*
Heart	*ubiq/ef1a*	*ubiq/18s*	*18s*	*rplp1*	*rplp1*	*actb*	*actb*	*ef1a*
Intestine	*actb/ubiq*	*g6pd/18s*	*ef1a*	*rplp1*	*rplp1*	*ef1a*	*tba1c*	*actb*
Spleen	*rplp1/ubiq*	*actb/ubiq*	*18s*	*tba1c*	*ef1a*	*rplp1*	*actb*	*18s*
Kidney	*ubiq/ef1a*	*rplp1/ef1a*	*rplp1*	*g6pd*	*b2m*	*tba1c*	*18s*	*ubiq*
Gill	*g6pd/actb*	*tba1c/ubiq*	*ubiq*	*rplp1*	*tba1c*	*ef1a*	*18s*	*actb*
Pooled tissues	*ubiq/rplp1*	*ubiq/18s*	*b2m*	*ef1a*	*18s*	*rplp1*	*ef1a*	*tba1c*

Stability ranking of individual reference genes in different tissues from Atlantic cod under control (stable temperature) or stress situation (temperature increase of 1?C/day for 5 days) conditions.

### Normfinder

**Figure 3:**
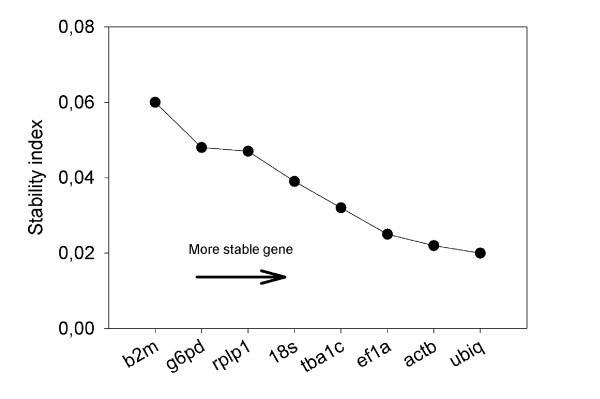
**Normfinder analysis of reference genes in liver**. Ranking of the reference genes stability of all 70 tissue samples (heart, spleen, head kidney, small intestine, gills and liver) from both groups. The analysis was also done after pooling the data from all tissues. According to the Normfinder software, a low average stability value indicates more stable expression.

Expression data from tissues were analyzed both separately and pooled. In liver tissue, *ubiq *and *actb *were the two most stable reference genes according to Normfinder (Figure 3). *ef1a *and *ubiq *were also most frequently among the the 3 most stable genes in the separate tissues (Table 3). However, when data from all tissues in both experimental groups were pooled and analyzed, *rplp1 *was the most stable gene.

**Table 3:** Normfinder ranking of gene stability in individual tissues.

**Liver**	*b2m*	*g6pd*	*rplp1*	*18s*	*tba1c*	*ef1a*	*actb*	*ubiq*
**Heart**	*b2m*	*g6pd*	*rplp1*	*actb*	*18s*	*ubiq*	*tba1c*	*ef1a*
**Intestine**	*rplp1*	*b2m*	*g6pd*	*ubiq*	*tba1c*	*18s*	*ef1a*	*actb*
**Spleen**	*tba1c*	*rplp1*	*g6pd*	*b2m*	*ubiq*	*actb*	*ef1a*	*18s*
**Kidney**	*tba1c*	*18s*	*rplp1*	*actb*	*g6pd*	*ubiq*	*b2m*	*ef1a*
**Gill**	*ef1a*	*rplp1*	*tba1c*	*actb*	*b2m*	*ubiq*	*18s*	*g6pd*
**Stability**								

Normfinder ranking of candidate reference genes from various tissues in Atlantic cod. Increasing stability towards the right side of the table.

### Bestkeeper

Bestkeeper performs a correlation analysis of Cp values and ranked the genes from pooled data from all samples in following order, from most stable to least stable; *actb>g6pd>rplp1>ubiq>ef1a>tba1c>18s>b2m*. As can be seen from Table 4 all reference genes investigated were positively correlated to each other and the highest correlations were found between *rplp1/g6pd *(r = 0,981) and *ef1a/g6pd *(r = 0,929). This method therefore ranked the reference genes somewhat different than geNorm and Normfinder, but *rplp1 *and *ubiq *were still among the four best suited reference genes.

**Table 4:** Bestkeeper analysis of reference genes from Atlantic cod.

*Pearson correlation coefficient (r)*								
	*rplp1*	*g6pd*	*tba1c*	*actb*	*b2m*	*ubiq*	*ef1a*	*18s*
**vs**.	HKG 1	HKG 2	HKG 3	HKG 4	HKG 5	HKG 6	HKG 7	HKG 8
***g6pd***	**0,981**	-	-	-	-	-	-	-
p-value	0,001	-	-	-	-	-	-	-
***tba1c***	0,685	0,688	-	-	-	-	-	-
p-value	0,029	0,028	-	-	-	-	-	-
***actb***	**0,865**	**0,885**	**0,853**	-	-	-	-	-
p-value	0,001	0,001	0,002	-	-	-	-	-
***b2m***	0,012	0,081	0,284	0,390	-	-	-	-
p-value	0,977	0,824	0,425	0,264	-	-	-	-
***ubiq***	**0,857**	**0,800**	0,765	**0,868**	0,297	-	-	-
p-value	0,002	0,005	0,010	0,001	0,405	-	-	-
***ef1a***	**0,922**	**0,929**	0,592	0,754	-0,147	0,707	-	-
p-value	0,001	0,001	0,071	0,012	0,686	0,022	-	-
***18s***	0,537	0,516	0,647	0,600	0,276	0,676	0,573	-
p-value	0,110	0,128	0,043	0,067	0,441	0,032	0,083	-
								
RANK	3	2	6	1	8	4	5	7
BestKeeper vs.	***rplp1***	***g6pd***	***tba1c***	***actb***	***b2m***	***ubiq***	***ef1a***	***18s***
coeff. of corr. [r]	0,950	0,954	0,816	0,960	0,265	0,912	0,871	0,683
p-value	0,001	0,001	0,004	0,001	0,458	0,001	0,001	0,030

Bestkeeper analysis of candidate reference genes from various tissues in Atlantic cod. Pair wise correlation analysis showing Pearson correlation coefficient (r) and the value of probability, p. Pair of genes with r > 0,80 in bold.

### Expression of heat shock proteins

The packages use different algorithms to determine the most stable reference genes. The selection of genes for further use is therefore a result of choosing the genes that overall got the highest ranking by all software packages. Four genes that were ranked high in stability by all three tools were *ef1a*, *rplp1*, *18s *and *ubiq*.

**Figure 4:**
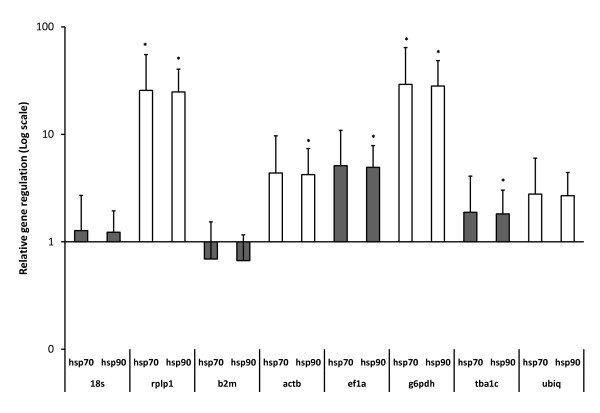
**REST analysis of relative *hsp70 *and *hsp90 *gene expression in cod liver after hyperthermic stress**. Results from REST analysis of target genes normalized against of the different reference genes identified in this study. (mean ± SE). Star above bar indicates significant change from control (p < 0.05).

To confirm that the fishes in the heat stress group actually experienced a stress reaction we analyzed the expression of two of the heat shock proteins (obtained from cod sequence databases). Alignment of the translated HSP70 sequence from which primers were constructed with similar proteins from human and other fishes (Additional file 2 and 3) revealed similarity to heat inducible HSP70's from other fishes [11]. The cod sequence grouped together with inducible HSP70's from several fish species and not with the cognate (constitutive) human HSPA8 protein [12]. In a similar fashion we aligned the cod HSP90 sequence with fish and human homologs and made a phylogenetic tree by maximum likelihood method as for HSP70 sequences (Additional file 4 and 5). Cod HSP90 grouped together with other inducible fish HSP90AA's, and not with the constitutive HSP90AB proteins [13]. The effect of hyperthermia on these transcripts was then analyzed by RT-qPCR using the each of the investigated reference genes as normalizers. Figure 4 shows that the two heat shock genes were induced relative to all normalizers, except *b2m*, the most unstable reference gene in liver, according to the Normfinder analysis (Table 3), indicating that this transcript was induced even more by hyperthermia.

## Discussion

Previous studies on hyperthermia in cod have shown that both acute and long term exposure to elevated temperatures induce stress responses in cod [14,15] including changes in gene expression [15]. Olsvik et al has investigated suitable reference genes for wild populations of Atlantic cod with focus on gene stability in fish living in contaminated areas. Using geNorm and Normfinder they found that *ubiq *(*ubiquitin) *and *rplp1 (called arp in their article) *was the most suitable reference genes for wild populations [1,16]. Similarly, during ontogeny in cod larva, *ubiq *(*ubiquitin) *was also found to be the most stable reference gene [16]. Optimally, genes from different biological pathways should be selected to minimize the effects of genes co-regulation [6]. The reference genes used in this trial were chosen on the basis of sequence availability for the cod genome and previous studies in cod [1] and other species [17-20]. If the genes chosen are included in the same biological pathway this may interfere with the analysis. This is because the software performs pair wise comparisons of gene expression across the various samples. Co-expressed genes will therefore most probably have similar expression patterns. If the genes are co-regulated in the same pathway geNorm tend to assign good score because it uses a pair wise comparison approach. This is important to keep in mind when interpreting the results. In this situation a model-based evaluation like Normfinder would be a better choice.

When comparing the results from geNorm, Normfinder and Bestkeeper, *rplp1 *and *ubiq *were the genes ranked with the highest expression stability by all the three methods. These results are in line with previously tested reference genes from Atlantic cod [1]. In six tissues from four different populations of cod, *ef1a, rplp1 *and *ubiq *(in addition to three ribosomal proteins) were found to be the most stable reference genes. The poor performance of the widely used *actb *and *g6pd *is in line with studies in other fish species [17,20,21].

However, it is important to take into consideration that reference genes that are stable in one species under one type of stress may be induced or repressed in other species during the same conditions [20]. Both *hsp70 *and *hsp90 *were induced relative to the most stable reference genes identified in this study. Although hyperthermia induced expression of *hsp70 *has been shown in many species [22,23], it has also previously been demonstrated that a rise in temperature of 1°C/day from 11 to 16.5°C does not increase western blot detectable hsp70 in Atlantic cod [24]. The reason for this discrepancy between protein and mRNA from two comparable studies is uncertain, but there are at least 2 isoforms of hsp70 where one is constitutive and one is stress induced [25].

## Conclusions

This is the first study that evaluates a suitable set of reference genes during hyperthermia in Atlantic Cod. From different types of software analyses at least 2-3 reference genes should be used and the most suitable reference genes under these experimental conditions were *18s, ef1a*, *rplp1 *and *ubiq*.

## Competing interests

The authors declare that they have no competing interests.

## Authors' contributions

IAA carried out the RT-qPCR analyses, performed the statistical analyses and drafted the manuscript. ALR participated in the sampling of the tissues, RNA isolation and RT-qPCR. HEK participated in planning of the study, was responsible for animal husbandry and experimental setup for the temperature study. TG conceived the study, participated in study design and coordination and carried out sequence alignments and drafted the manuscript. All authors read and approved the final manuscript.

## Supplementary Material

Additional file 1 **Determination of optimal number of genes for normalization**. Pairwise variation analysis (V_n/n+1_) between two sequential normalization factors (reference genes) NF_n _and NF_n+1 _to determine the optimal number of reference genes for accurate normalization in control group (Panel A) and stress group (Panel B). Low pairwise variation (< 0.15) indicates no requirement for additional reference genes. Click here for file

Additional file 2 **Alignment of partial HSP70 sequences**. Alignment of partial HSP70 sequences from various fish and mammals. Dots indicate identity. Sequences are from Danio rerio (Dre) BC056709.1, Caarassius auratus (Cau) AB = 92839.2, Cyprinus carpio (Cca) AY120894.1, Oreochromis niloticus (Oni) FJ213839.1, Xiphophorus maculatus (Xma) AB062114.1 = HSP70-2 and AB062113.1 = HSP70-1, Oncorhynchus mykiss (Omy) AB062281.1, Salmo salar (Ssa) BT046112.1, Paralichthys olivaceus (Pol) DQ662230.1, Homo sapiens (Hsa) AAH07276.2. = HSP1A, and ENSP 00000227378 = HSPA8. Click here for file

Additional file 3 **Phylogenetic tree of HSP 70 sequences**. Phylogenetic tree showing relationship between several fish and 2 mammalian HSP70 proteins using maximum likelihood method. Bootstrap values are shown on the branches and scale for branch length (0.05 substitutions/site). Sequences are from Danio rerio (Dre) BC056709.1, Caarassius auratus (Cau) AB = 92839.2, Cyprinus carpio (Cca) AY120894.1, Oreochromis niloticus (Oni) FJ213839.1, Xiphophorus maculatus (Xma) AB062114.1 = HSP70-2 and AB062113.1 = HSP70-1, Oncorhynchus mykiss (Omy) AB062281.1, Salmo salar (Ssa) BT046112.1, Paralichthys olivaceus (Pol) DQ662230.1, Homo sapiens (Hsa) AAH07276.2. = HSP1A, and ENSP 00000227378 = HSPA8. Click here for file

Additional file 4 **Alignment of partial HSP90 sequences**. Alignment of partial HSP90 sequences from various fish and mammals. Dots indicate identity. Sequences are from Paralichthys olivaceus (Pol) Dq66233.1 = HSP90AA and AY214170.1 = HSP90AB, Danio rerio (Dre) NM001045073.1 and AF042108.1 = HSP90AB, Solea senegalensis (Sse) AB367526.1 and AB367527.1 = HSP90AB, Salmo salar (Ssa) NM 001173702.1 = HSP90AA and NM_001123532.1 = HSP90AB, Gadus morhua (Gmo) ES783928, Homo sapiens (Hsa) DC303876.1 = HSP90AA and BC004928.1 = HSP90AB. Click here for file

Additional file 5 **Phylogenetic tre of HSP90 sequences**. Phylogenetic tree showing relationship between several fish and 2 mammalian HSP90 proteins using maximum likelihood method. Bootstrap values are shown on the branches and scale for branch length (0.035 substitutions/site). Sequences are from Paralichthys olivaceus (Pol) Dq66233.1 = HSP90AA and AY214170.1 = HSP90AB, Danio rerio (Dre) NM001045073.1 and AF042108.1 = HSP90AB, Solea senegalensis (Sse) AB367526.1 and AB367527.1 = HSP90AB, Salmo salar (Ssa) NM 001173702.1 = HSP90AA and NM_001123532.1 = HSP90AB, Gadus morhua (Gmo) ES783928, Homo sapiens (Hsa) DC303876.1 = HSP90AA and BC004928.1 = HSP90AB. Click here for file
