# Supplementary figures and images for: Validation of reference genes for quantitative RT-qPCR studies of gene expression in Atlantic cod (Gadus morhua l.) during temperature stress
Source: BMC Res Notes. 2011 Apr 5;4:104. doi: 10.1186/1756-0500-4-104 (PMC3080820; doi:10.1186/1756-0500-4-104)

**A**

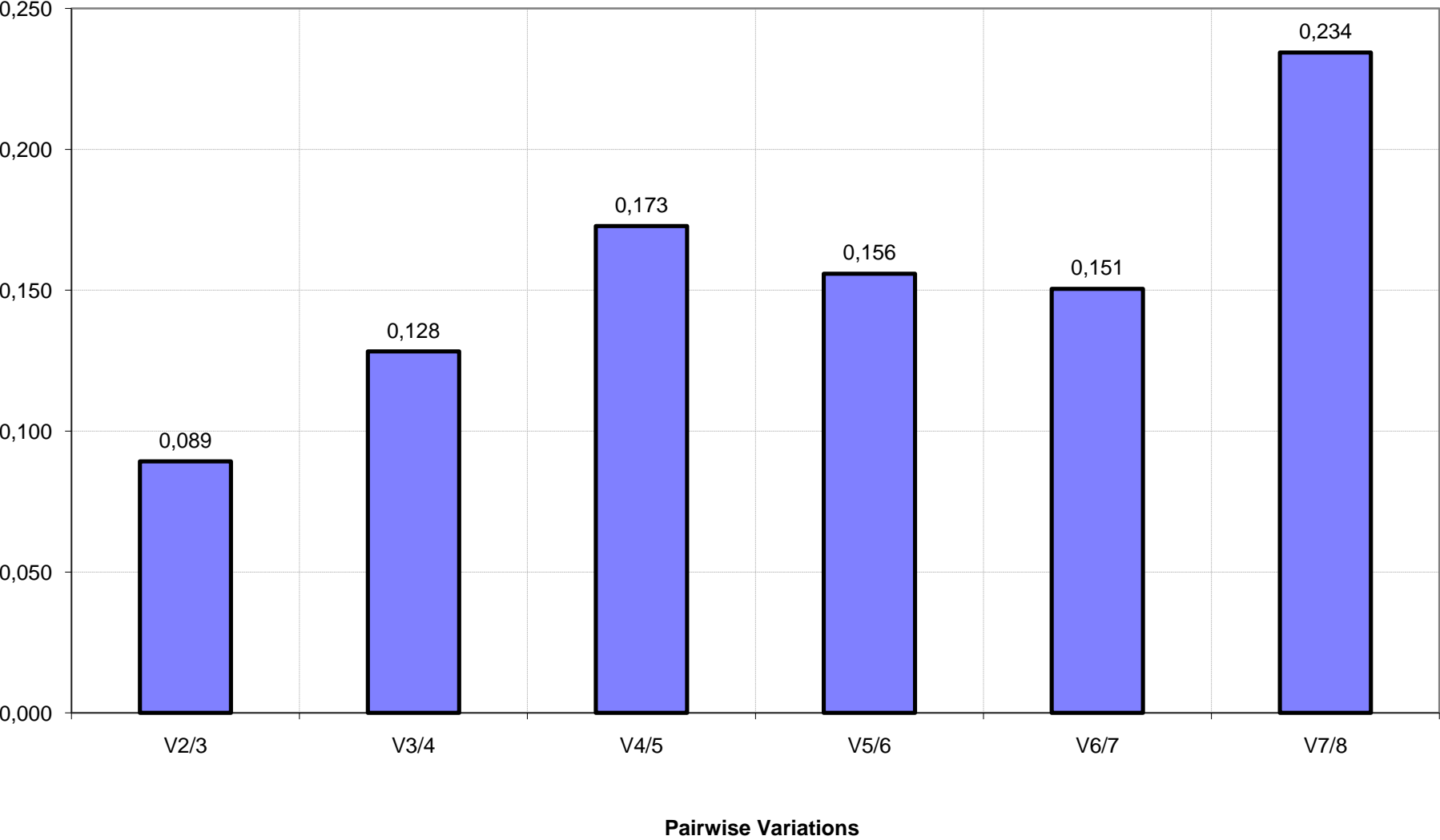

**B**

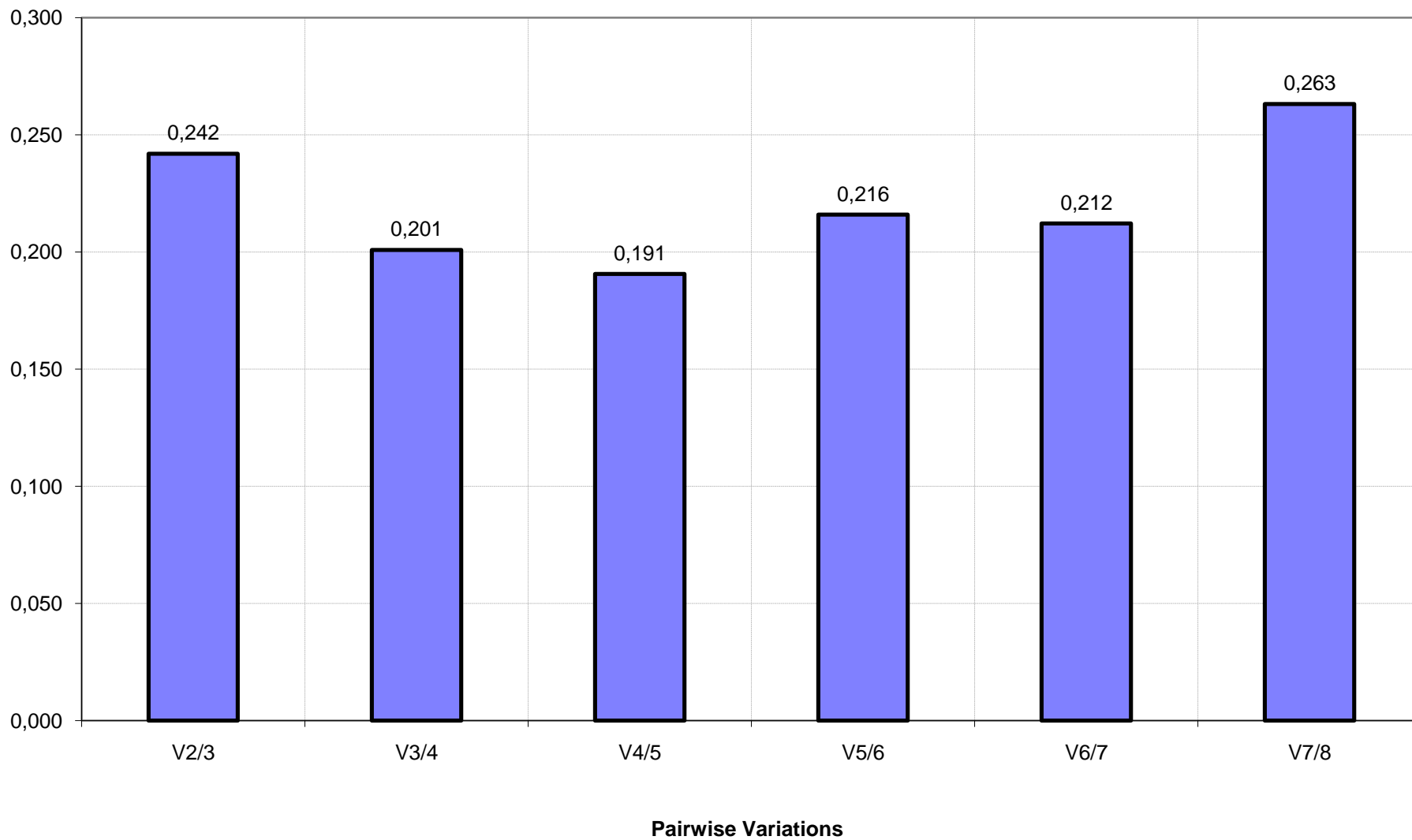

Supplement: Additional file 1 — Determination of optimal number of genes for normalization. Pairwise variation analysis (Vn/n+1) between two sequential normalization factors (reference genes) NFn and NFn+1 to determine the optimal number of reference genes for accurate normalization in control group (Panel A) and stress group (Panel B). Low pairwise variation (< 0.15) indicates no requirement for additional reference genes. [file 1756-0500-4-104-S1.PDF]

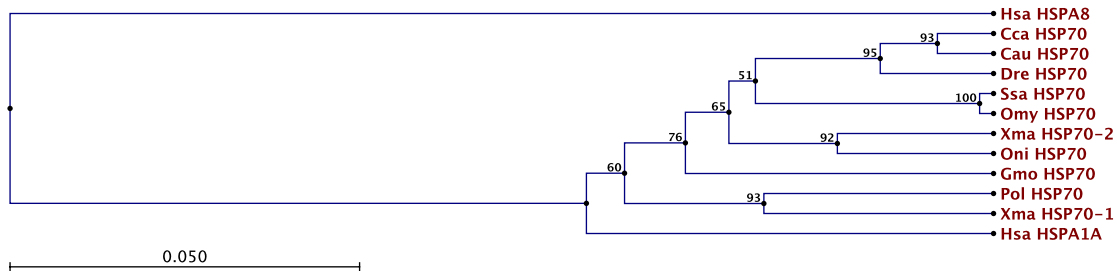

Additional file 3

Supplement: Additional file 3 — Phylogenetic tree of HSP 70 sequences. Phylogenetic tree showing relationship between several fish and 2 mammalian HSP70 proteins using maximum likelihood method. Bootstrap values are shown on the branches and scale for branch length (0.05 substitutions/site). Sequences are from Danio rerio (Dre) BC056709.1, Caarassius auratus (Cau) AB = 92839.2, Cyprinus carpio (Cca) AY120894.1, Oreochromis niloticus (Oni) FJ213839.1, Xiphophorus maculatus (Xma) AB062114.1 = HSP70-2 and AB062113.1 = HSP70-1, Oncorhynchus mykiss (Omy) AB062281.1, Salmo salar (Ssa) BT046112.1, Paralichthys olivaceus (Pol) DQ662230.1, Homo sapiens (Hsa) AAH07276.2. = HSP1A, and ENSP 00000227378 = HSPA8. [file 1756-0500-4-104-S3.PDF]

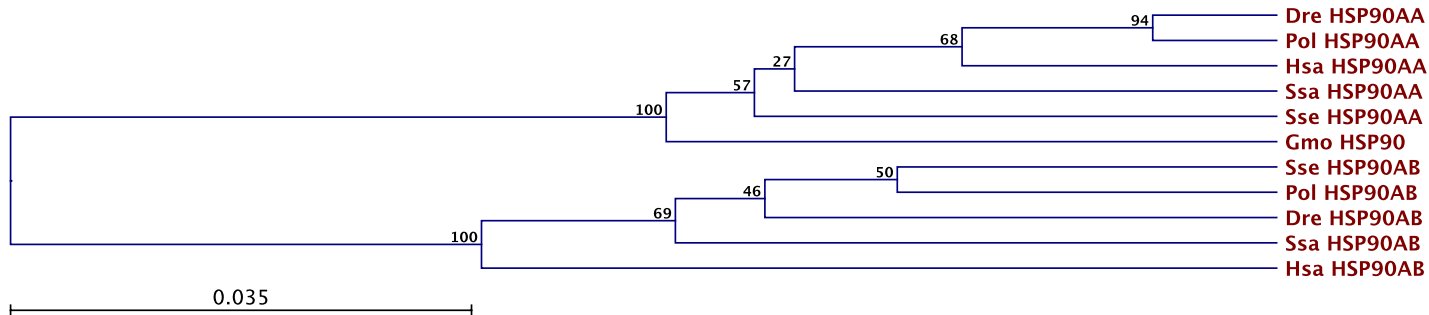

Additional file 5

Supplement: Additional file 5 — Phylogenetic tre of HSP90 sequences. Phylogenetic tree showing relationship between several fish and 2 mammalian HSP90 proteins using maximum likelihood method. Bootstrap values are shown on the branches and scale for branch length (0.035 substitutions/site). Sequences are from Paralichthys olivaceus (Pol) Dq66233.1 = HSP90AA and AY214170.1 = HSP90AB, Danio rerio (Dre) NM001045073.1 and AF042108.1 = HSP90AB, Solea senegalensis (Sse) AB367526.1 and AB367527.1 = HSP90AB, Salmo salar (Ssa) NM 001173702.1 = HSP90AA and NM_001123532.1 = HSP90AB, Gadus morhua (Gmo) ES783928, Homo sapiens (Hsa) DC303876.1 = HSP90AA and BC004928.1 = HSP90AB. [file 1756-0500-4-104-S5.PDF]
