# Supplementary material for: Validation of reference genes for quantitative RT-qPCR studies of gene expression in Atlantic cod (Gadus morhua l.) during temperature stress
Source: BMC Res Notes. 2011 Apr 5;4:104. doi: 10.1186/1756-0500-4-104 (PMC3080820; doi:10.1186/1756-0500-4-104)
Supplement: Additional file 2 — Alignment of partial HSP70 sequences. Alignment of partial HSP70 sequences from various fish and mammals. Dots indicate identity. Sequences are from Danio rerio (Dre) BC056709.1, Caarassius auratus (Cau) AB = 92839.2, Cyprinus carpio (Cca) AY120894.1, Oreochromis niloticus (Oni) FJ213839.1, Xiphophorus maculatus (Xma) AB062114.1 = HSP70-2 and AB062113.1 = HSP70-1, Oncorhynchus mykiss (Omy) AB062281.1, Salmo salar (Ssa) BT046112.1, Paralichthys olivaceus (Pol) DQ662230.1, Homo sapiens (Hsa) AAH07276.2. = HSP1A, and ENSP 00000227378 = HSPA8. [file 1756-0500-4-104-S2.PDF]

```
Cau HSP70    IANDQGNRTT PSYVAFTDTE RLIGDAAKNQ VAMNPNNTVF DAKRLIGRKF EDPVVQSDMK 60
Cca HSP70    .......... .......... .......... .......... .......... D......... 60
Dre HSP70    .......... .......... .......... .......... ........R. D......... 60
Oni HSP70    .......... .......... .......... ..L..S.... .......... DE....A... 60
Xma HSP70-2  .......... .......... .......... ..L..S.... .......... .E....A... 60
Omy HSP70    .......... .......... .......... .......... .......... N.Q...A... 60
Ssa HSP70    .......... .......... .......... .......... .......... N.Q...A... 60
Gmo HSP70    .....C.... .......... ....G..... .....T.... .......... D.A...A... 60
Xma HSP70-1  .......... .......... .......... .....T..I. ........R. D.L....... 60
Pol HSP70    .......... .......... .......... .....T..I. .......... N.SI...... 60
Hsa HSPA1A   .......... .......... .......... ..L..Q.... .......... G......... 60
Hsa HSPA8    ---------- ---------- ---------- -....T.... ........R. D.A....... 29

Cau HSP70    HWSFQVISDG GKPKVQVEYK GENKTFYPEE ISSMVLVKMK EIAEAYLGQK VTNAVITVPA 120
Cca HSP70    ......V... .......... .......... .......... .......... .......... 120
Dre HSP70    ....K.V... .....A..H. ......N... .......... .......... .......... 120
Oni HSP70    ..P.K..... ....IR.... ..D.A..... .......... .......... .S........ 120
Xma HSP70-2  ..P.E.L... .R..I..... ....A.F... .......... ........H. .S........ 120
Omy HSP70    ..P.K.V... .......D.. ....S.N... .........R .......... .S........ 120
Ssa HSP70    ..P.K.V... .......D.. ....S.N... .........R .......... .S........ 120
Gmo HSP70    ..P.T.M..A ....I...H. ....S..... V......... .......... .S........ 120
Xma HSP70-1  L.P.K..N.N .......... ..I...C... .........R .....F...R .S........ 120
Pol HSP70    L.P.K....N .......... ..T.A..... .......... .......... .S........ 120
Hsa HSPA1A   ..P....N.. D......S.. ..T.A..... ......T... ........YP .......... 120
Hsa HSPA8    ..P.M.VN.A .R........ ..T.S..... V.....T... ........KT .....V.... 89

Cau HSP70    YFNDSQRQAT KDAGVIAGLN VLRIINEPTA AAIAYGLDKG KASERNVLIF DLGGGTFDVS 180
Cca HSP70    .......... .......... ..EN...... .......... .......... .......... 180
Dre HSP70    .......... .......... .......... .......... .S........ .......... 180
Oni HSP70    .......... .......... .......... .......... .SG....... .......... 180
Xma HSP70-2  .......... .......... .......... .......... .SG....... .......... 180
Omy HSP70    .......... .......... .......... ......M... .SR....... .......... 180
Ssa HSP70    .......... .......... .......... ......M... MSR....... .......... 180
Gmo HSP70    .......... .......... .......... .......... .S........ .......... 180
Xma HSP70-1  .......... ......S... .......... .......... .RG....... .......... 180
Pol HSP70    .......... ......S... I......... .......... .RG....... .......... 180
Hsa HSPA1A   .......... .......... .......... ........RT GKG....... .......... 180
Hsa HSPA8    .......... ....T..... .......... .........K VGA....... .......... 149

Cau HSP70    ILTIEDGIFE VKATAGDTHL GGEDFDNRMV NHFVEEFKRK HKKDISQNKR ALRRLRTACE 240
Cca HSP70    .......... .......... .......... .......... .......... .......... 240
Dre HSP70    .......... .......... .......... .......... .......... .......... 240
Oni HSP70    .......... ..S....... .......... .......... .......... .......... 240
Xma HSP70-2  V......... .......... .......... .......... .......... .......... 240
Omy HSP70    .......... .......... ........L. S......... .......... .......... 240
Ssa HSP70    .......... .......... ........L. S......... .......... .......... 240
Gmo HSP70    .......... ..S....... ........L. D......... F......... .......... 240
Xma HSP70-1  .......... ..S....... .......... S......... .......... .V........ 240
Pol HSP70    .......... .......... .......... S..L...... Y......... .V........ 240
Hsa HSPA1A   ....D..... .......... ........L. .......... .......... .V........ 240
Hsa HSPA8    .......... ..S....... .......... ...IA..... ......E... .V........ 209

Cau HSP70    RAKRTLSSSS Q- 251
Cca HSP70    .......... .- 251
Dre HSP70    .......... .- 251
Oni HSP70    .......... .A 252
Xma HSP70-2  .......... .- 251
Omy HSP70    .......... .- 251
Ssa HSP70    .......... .- 251
Gmo HSP70    .......... -- 250
Xma HSP70-1  .........T .A 252
Pol HSP70    .........T .- 251
Hsa HSPA1A   .........T .A 252
Hsa HSPA8    .........T .- 220
```

Additional file 2
