# Supplementary material for: Validation of reference genes for quantitative RT-qPCR studies of gene expression in Atlantic cod (Gadus morhua l.) during temperature stress
Source: BMC Res Notes. 2011 Apr 5;4:104. doi: 10.1186/1756-0500-4-104 (PMC3080820; doi:10.1186/1756-0500-4-104)
Supplement: Additional file 4 — Alignment of partial HSP90 sequences. Alignment of partial HSP90 sequences from various fish and mammals. Dots indicate identity. Sequences are from Paralichthys olivaceus (Pol) Dq66233.1 = HSP90AA and AY214170.1 = HSP90AB, Danio rerio (Dre) NM001045073.1 and AF042108.1 = HSP90AB, Solea senegalensis (Sse) AB367526.1 and AB367527.1 = HSP90AB, Salmo salar (Ssa) NM 001173702.1 = HSP90AA and NM_001123532.1 = HSP90AB, Gadus morhua (Gmo) ES783928, Homo sapiens (Hsa) DC303876.1 = HSP90AA and BC004928.1 = HSP90AB. [file 1756-0500-4-104-S4.PDF]

```
Pol HSP90AA  RVFIMDNCDE LIPEYLNFIR GVVDSEDLPL NISREMLQQS KILKVIRKNL VKKCLELFTE 60
Dre HSP90AA  .......... .........K .......... .......... .......... .......... 60
Hsa HSP90AA  ........E. .......... .......... .......... .......... .......... 60
Ssa HSP90AA  .........D .........K .......... .......... .......... ....I..... 60
Sse HSP90AA  ........E. .........K .......... .......... .......... ....M...I. 60
Gmo HSP90    .......... .M....D..K .......... .......... .......... .....D.... 60
Pol HSP90AB  ........E. ........V. .......... .......... .........I ........A. 60
Sse HSP90AB  ........E. ........V. .......... .......... .........I ........A. 60
Dre HSP90AB  ........E. .......... .......... .......... .........I ........A. 60
Ssa HSP90AB  ......S.E. ........V. .......... .......... .........I ....M...G. 60
Hsa HSP90AB  ......S... .......... .......... .......... .........I ........S. 60

Pol HSP90AA  LAEDKDNYKK YYEQFSKNIK LGIHEDSQNR KKLSELLRYY TSSSGDEMVS LKDYVTRMKD 120
Dre HSP90AA  .......... .......... .......... .......... ..A....... .......... 120
Hsa HSP90AA  .....E.... F......... .......... .......... ..A....... ....C....E 120
Ssa HSP90AA  .S...E.... .......... .......... .R..DM.... ..A....... .........E 120
Sse HSP90AA  .S........ F......... .......... ....DM.... ..A.....I. M....S...E 120
Gmo HSP90    ..D..E.... C......... ......A... .......... ..TT...... .....S...E 120
Pol HSP90AB  .....E.... F..G...... .......... .........Q S.Q....ST. .TE.LS...E 120
Sse HSP90AB  ...A.E...S F..A...... .......... .........H S.Q.....S. .TE.IS.... 120
Dre HSP90AB  .......... F.DA....L. ......C... .........Q S.Q.....T. .TE..S...E 120
Ssa HSP90AB  ....RE..N. F.DG....L. .......... .........H S.Q....LT. .TE.L..... 120
Hsa HSP90AB  .....E.... F..A....L. .......T.. RR.......H ..Q.....T. .SE..S...E 120

Pol HSP90AA  NQKHIYYITG ETKDQVANSA FVERLRKAGL EVIYMIEPID E 161
Dre HSP90AA  T......... .......... .......... .......... . 161
Hsa HSP90AA  .......... .......... .......H.. .......... . 161
Ssa HSP90AA  T......... ..R....... .......... .......... . 161
Sse HSP90AA  .......... .......... .......... .......... . 161
Gmo HSP90    .......... .......... .......... .......... . 161
Pol HSP90AB  ...S...... .S........ ....V..R.F ..L..T.... . 161
Sse HSP90AB  ...A...... .S........ ....V..R.F ..L..T.... . 161
Dre HSP90AB  ...S...... .S.....H.. ....VC.R.F ..L..T.... . 161
Ssa HSP90AB  ...S...... .S........ ....V..R.F ..L..T.... . 161
Hsa HSP90AB  T..S...... .S.E...... ....V..R.F ..V..T.... . 161
```

Additional file 4
